# Benign and Malignant Parotid Gland Tumors: Insights from a Five-Year Northeast Romanian Population

**DOI:** 10.3390/jcm14197087

**Published:** 2025-10-08

**Authors:** Loredana-Beatrice Ungureanu, Cristina-Mihaela Ghiciuc, Victor Vlad Costan, Carmen Ungureanu, Victor Ianole, Delia-Gabriela Ciobanu Apostol

**Affiliations:** 1 Department of Morpho-Functional Sciences I—Pathology, Grigore T. Popa University of Medicine and Pharmacy, 700115 Iasi, Romania; loredana.ungureanu@umfiasi.ro (L.-B.U.); carmen.ungureanu@umfiasi.ro (C.U.); ianole.victor@umfiasi.ro (V.I.); delia.ciobanu@umfiasi.ro (D.-G.C.A.); 2 Department of Morpho-Functional Sciences II—Pharmacology and Clinical Pharmacology, Faculty of Medicine, Grigore T. Popa University of Medicine and Pharmacy of Iasi, 16 Universitatii Street, 700115 Iasi, Romania; 3 Pediatric Emergency Hospital Sf Maria, 700887 Iasi, Romania; 4 Department of Surgery, Faculty of Dental Medicine, Grigore T. Popa, 16 Universitatii Str., 700115 Iasi, Romania; victor.costan@umfiasi.ro; 5 Pathology Department, Saint Emergency Spiridon University Hospital, 700111 Iasi, Romania

**Keywords:** parotid gland benign tumors, parotid gland malignant tumors, parotid neoplasm diagnosis, parotid gland histologic subtypes, demographic, salivary gland surgery

## Abstract

**Background**: The majority of parotid gland tumors are benign, while malignant forms are uncommon, affecting fewer than 1 in 100,000 individuals. The main challenge resides in the histopathological complexity and the clinical overlap between benign and malignant parotid tumors, which frequently results in misdiagnosis. **Aim**: The objective of this research was to evaluate the clinical and histopathological characteristics of parotid gland tumors at a Romanian healthcare center. **Materials and methods**: A five-year retrospective study was conducted, with the inclusion criterion being the presence of complete clinical, pathological, and surgical records. **Results**: Of 156 patients included in the study, 67.3% were found to have benign lesions (male/female ratio 1.14:1), and there was a slight male predominance (53.3%). Partial parotidectomy was the most common surgical intervention for benign parotid tumors (59.6%), whereas total parotidectomy was predominantly indicated for malignant tumors, with facial nerve sacrifice occurring in 20% of cases to ensure complete tumor excision. Patients with benign tumors were found to be younger. Malignant tumors were commonly diagnosed at stage III (36.4%), indicative of more advanced disease at the time of diagnosis. Clinical diagnosis showed a high specificity of 96.9%, indicating high accuracy in malignancy suspicion, yet the sensitivity of 56% indicates that a significant number of malignancies were not detected during the initial evaluation. Tumor size was found to be influenced by gender and correlated with surgical methods, suggesting that patient characteristics and tumor biology may impact surgical strategy. **Conclusions**: This retrospective study highlights differences in gender, tumor size, and surgical approach between benign and malignant parotid gland tumors, offering valuable contributions in terms of diagnostic accuracy and treatment patterns despite a limited number of malignant cases.

## 1. Introduction

Salivary gland tumors predominantly develop in the parotid gland, accounting for about 85% of all salivary tumors [1,2]. Overall, salivary gland tumors represent 3–6% of all head and neck tumors [3]. Their incidence remains generally low, with fewer than 15 cases estimated per 100,000 individuals per year [3]. Despite their varied histological types and evolving WHO classifications [4], these tumors have distinct clinical behavior. The main challenge lies in the histopathological complexity of parotid tumors, which have multiple subtypes [4,5], and in the high degree of interobserver variability, particularly in cytological assessment [6]. There is also frequent misdiagnosis of rare entities [7], and an absence of reliable molecular biomarkers for prognosis or treatment guidance [8].

Most parotid gland tumors are benign (85%) [9], with pleomorphic adenoma being the most common parotid gland tumor, representing 60–70% of cases [10,11,12]. Despite its benign nature, pleomorphic adenoma sometimes has the potential to recur, metastasize, or undergo malignant transformation into carcinoma ex pleomorphic adenoma [1,13]. Warthin’s tumor is the second most common benign parotid gland tumor, which is characterized by a high incidence in males and an association with smoking [14]. Only about 15% of cases are classified as malignant [9]. Some benign tumors can recur or undergo malignant transformation. However, specific malignant subtypes such as acinic cell carcinoma have favorable prognoses, with long-term survival sometimes exceeding 20 years [15,16]. Malignant neoplasms of the parotid gland may exhibit characteristics that closely resemble those of benign tumors. Consequently, differentiating between benign and malignant parotid tumors, both clinically and histologically, is challenging and poses considerable difficulty [17]. Mucoepidermoid carcinoma is often cited as the most common malign type [9,18], accounting for up to 50% of documented cases in some studies [18].

The clinical overlap between benign and malignant parotid tumors often leads to misdiagnosis because some malignant tumors may present with slow growth and no adjacent tissue infiltration [17]. Preoperative biopsy is generally avoided due to risks of tumor spillage, facial nerve injury, and salivary fistula formation. Fine needle aspiration cytology is useful but remains unreliable in certain malignant cases. Definitive diagnosis is usually histopathological after surgery [19]. The standard treatment is surgical excision, most commonly via partial (superficial) or total parotidectomy with facial nerve preservation. However, malignant tumors often require more extensive resection [9]. Complications include facial nerve injury, Frey syndrome, and tumor rupture with risk of recurrence [1,10,20]. In benign tumors such as pleomorphic adenoma, incomplete excision increases the risk of recurrence [12], and nerve preservation is especially challenging in malignant or recurrent cases [21,22]. The parotid gland is also vulnerable to metastases from squamous cell carcinoma, melanoma, and rarely from distant primaries such as the breast, kidney, or pancreas [23]. There are still no predictors of recurrence or malignant transformation in pleomorphic adenoma [24], and parotid carcinoma has no specific targeted therapy, despite limited data on genetic alterations [25].

One study compared extracapsular dissection and superficial parotidectomy in pleomorphic adenoma [1]. Four reports on recurrent pleomorphic adenoma identified differences in age, gender, and tumor size between recurrent and nonrecurrent cases [10,11,26,27]. Factors predisposing to tumor rupture were described by Grasso et al. [12], while Andreasen et al. [28] reported on incidence, recurrence, and malignant transformation. Rajasekaran et al. [18] compared one-year survival and overall survival in mucoepidermoid carcinoma. Squamous cell carcinoma was analyzed with respect to frequency and five-year survival [29], as well as primary versus recurrent cases [30]. Risk factors and five-year survival in acinic cell carcinoma were examined by Scherl et al. [31]. Hu et al. [32] compared widely invasive carcinoma ex pleomorphic adenoma with intracapsular and minimally invasive subtypes. The prognostic influence of clinical and pathological features in myoepithelial carcinoma was investigated by Wang et al. [33]. Three studies addressed metastases: one based on histopathology [23] and two including survival analysis [34,35]. Finally, two studies assessed parotid lymphoma: one descriptive study [36] and one study with survival analysis [37].

The aim of this study was to analyze the clinical, demographic, and histopathological characteristics of parotid gland tumors diagnosed over a five-year period at a single Romanian medical center, with the goal of improving diagnostic accuracy, characterizing common presenting symptoms, and assessing treatment effectiveness to better manage these tumors.

## 2. Materials and Methods

### 2.1. Study Population

A retrospective analysis was conducted on all excision specimens for histopathologic evaluation from patients diagnosed with parotid gland tumor and admitted to the Oral and Maxillofacial Surgery Clinic at the Saint Spiridon Emergency University Hospital in Iași for a period of five years. Demographic and clinical data were obtained from the medical reports of these patients

This study was conducted in accordance with the Declaration of Helsinki and approved by the Ethics Committee of the Saint Spiridon Emergency University Hospital in Iași (protocol code 87 and date of approval 15 April 2025). Informed consent was obtained from each patient upon their admission to the hospital.

### 2.2. Data Description

Histopathological diagnoses were obtained from the records of the Pathological Department and reevaluated to establish the morphological criteria important for the diagnosis of these tumors. All slides were stained with hematoxylin–eosin and interpreted by two histopathologist (DGCA and LBU). All cases were reevaluated histopathologically and reclassified according to the WHO Classification of Head and Neck Tumors, 5th Edition, 2022 [4]. Cohen’s Kappa coefficient was calculated to evaluate the level of agreement. The results indicated a moderate level of agreement: κ = 0.40, *p* < 0.001.

The inclusion criteria for the study were patients with the histopathological diagnosis of parotid benign tumor or primary and secondary malignant parotid tumors. The exclusion criteria were lack of complete clinical–morphological data. Ultimately, only 156 cases with complete clinical data were included in the analysis.

Data were analyzed according to age at diagnosis, gender, place of origin, tumor size and location, clinical and histopathological diagnosis, and type of surgical intervention.

### 2.3. Statistical Analysis

All statistical analyses were conducted using SPSS version 26. Descriptive statistics (counts, percentages, means, and standard deviations where appropriate) were computed to summarize the study variables. Associations between categorical variables (such as sex, age groups, residence, treatment type, and evolution and size categories (<2 cm, 2–4 cm, >4 cm)) were examined, primarily using chi-square tests of independence. When more than 20% of the expected category count were below 5, Fisher’s exact test was applied as a more reliable alternative. The strength of significant associations was assessed using Cramer’s V (V), interpreted according to conventional thresholds (small = 0.10, medium = 0.30, large = 0.50). To further explore predictors of outcomes, binary logistic regression models were employed, allowing for the estimation of odds ratios (ORs) with 95% confidence intervals (CIs). Sensitivity and specificity were calculated using *VassarStats: Clinical Research Calculators* (http://vassarstats.net/ (accessed on 16 July 2025)). All tests were two-sided, and statistical significance was defined as *p* < 0.05.

## 3. Results

### 3.1. Study Sample Overview

**Figure 1:**
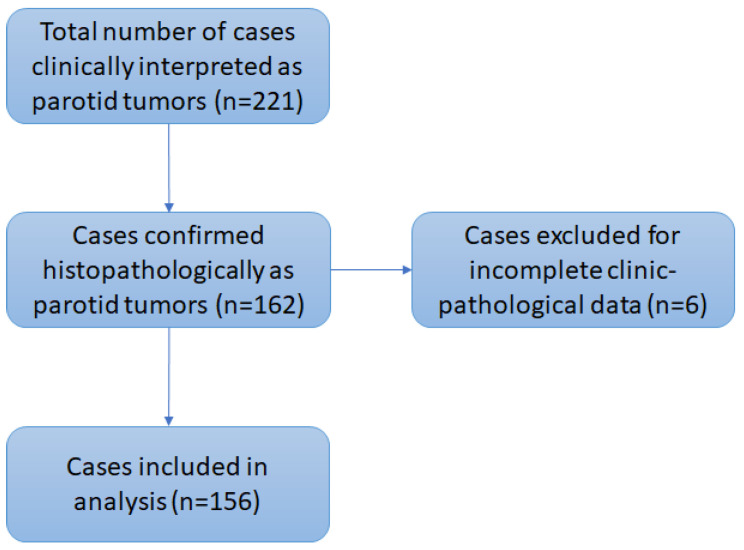
Case selection for analysis.

Out of a total of 221 cases with a clinical diagnosis of benign or malignant parotid tumors, only 156 cases were histologically confirmed as true parotid tumors (Figure 1). Preoperative biopsy was performed in 14 cases. In five cases, the clinical diagnosis was established after examination of the oral cavity for dental purposes.

Of the 156 cases with histopathologically confirmed parotid tumor diagnosis, there were 105 benign tumors (67.3% of all tumors). Of the 51 malignant tumors (32.7% of all tumors), 25 were primary tumors and 26 (51% of malignant tumors) were metastatic tumors (Table 1). The overall ratio of benign to malignant tumors in our study was 2.58:1.

In the present study, the most common benign tumor was pleomorphic adenoma (74 cases—47.4% of all tumors and 70.5% of benign tumors), and the most common malignant tumor was acinic cell carcinoma (6 cases—3.8% of all tumors and 24% of primary malignant tumors). Metastases were mainly represented by squamous cell carcinoma (55.5%), poorly differentiated adenocarcinoma (27.8%), and melanoma metastases (11.1%), all originating from tumors in the neighboring area.

The overall male-to-female ratio was 1.14:1. For benign tumors, the male-to-female ratio was 1.07:1, while for malignant tumors it was 1.23:1, indicating a slight predominance of males in both categories. Warthin tumors and squamous cell carcinomas occurred predominantly in male patients. In contrast, pleomorphic adenoma was more common in women and was diagnosed at a younger average age compared to Warthin tumors and malignant neoplasms.

### 3.2. Demographics

The characteristics of 221 cases with clinical diagnosis of parotid tumors are shown in Table 1, most of them (*n* = 161; 72.9%) being benign tumors, less being malignant tumors (n = 60; 27.2%). The male-to-female ratio was 1.12:1, while the rural-to-urban ratio was 1:0.90.

### 3.3. Tumor Distribution (Benign vs. Malignant)

The cohort of cases clinically diagnosed as parotid tumors was divided into two groups: benign tumors (161 cases) and malignant tumors (60 cases). Of benign tumors, 54.7% occurred in females, while 73.3% of malignant tumors occurred in males. Just over half of the sample (56%) was from an urban area in the case of benign tumors and from a rural area in the case of malignant tumors. Benign tumors most commonly occurred in the 41–70 age group, while malignant tumors occurred in the 51–80 age group most commonly.

### 3.4. Clinicopathological Characteristics of Benign Tumors

A total of 105 benign tumors were selected for the analysis. The mean age was 50.3 ± 15.7, ranging between 13 and 82 years old, and the mean size was 3.4 ±1.8 cm, ranging between 1 and 10.5 cm. In the present study, pleomorphic adenoma showed two incidence peaks, in the age ranges of 31–40 years and 51–60 years. Basal cell adenoma and Warthin tumor had a peak incidence within the age of 61 to 70 years. There was a statistically significant relationship between age group and diagnosis (*p* = 0.014), with a moderate association (V = 0.40). A chi-square test of independence was conducted to examine the association between sex and diagnosis (histopathological diagnosis). The results revealed a significant relationship between sex and diagnosis (*p* < 0.001) and moderate positive association (V = 0.41). Significantly more females than expected were diagnosed as pleomorphic adenoma (adjusted residual = 3.6), while males were significantly more likely to have Warthin tumor (adjusted residual = 4.1). While partial parotidectomy was the most common surgical approach, there were only two cases of pleomorphic adenoma and basal cell adenoma treated with total resection, and three cases of Warthin tumor treated with total resection and facial nerve preservation. The data are detailed in Table 2. In benign tumors, postoperative cavity plasty was performed in 83 patients with sternocleidomastoid muscle, in 32 patients with superficial musculoaponeurotic system (SMAS) flap, and in 1 patient with the posterior belly of the digastric muscle. No significant relation was found between histopathologic diagnosis in terms of residence (*p* = 0.070, effect size: V = 0.217), hospitalization length (*p* = 0.494, effect size: V = 0.193), or treatment (*p* = 0.054, effect size: V = 0.273).

### 3.5. Clinicopathological Characteristics of Malignant Tumors

Among the 51 malignant tumors, the mean patient age was 65.4 ± 15.5 years (range: 20–91 years), and the mean tumor size was 4.4 ± 2.5 cm (range: 1–13 cm).

Malignant tumors were mainly represented by primary tumors such as acinic cell carcinoma, carcinoma ex pleomorphic adenoma, adenocarcinoma NOS (not otherwise specified), adenoid cystic carcinoma, squamous cell carcinoma, basal cell adenocarcinoma, lymphoma, myoepithelial carcinoma, and mucoepidermoid carcinoma, but there were also intraparotid metastases. Tumor removal was performed by partial parotidectomy in 17 cases (33.3%), total parotidectomy with preservation of the facial nerve in 7 patients (6.7%), and total parotidectomy with sacrifice of the facial nerve in 16 cases (31.4%), mainly in the case of metastases in the surrounding area.

In 19 patients, the facial nerve was completely sacrificed, and in 3 cases, only its branches invaded by the tumor process were sacrificed. Loco-regional lymph node dissections were performed in 27 of these patients. Of the 33 malignant tumors with postoperative defects, 22 cases were reconstructed using a sternocleidomastoid muscle flap, while 8 cases were treated with SMAS and 3 cases with a latissimus dorsi FREE flap.

All lymphomas developed in parotid glands were non-Hodgkin lymphomas, one of them was lymphocytic lymphoma, and one was a large B cell lymphoma.

A chi-square test of independence was performed, and no significant differences among histological types in terms of age group (*p* = 0.811, effect size: V = 0.263), gender (*p* = 0.488, effect size: V = 0.097), residence (rural/urban) (*p* = 0.645, effect size: V = 0.065), tumor size (*p* = 0.170, effect size: V = 0.277), type of surgery (*p* = 0.401, effect size: V = 0.242), or clinical outcome at discharge (*p* = 0.289, effect size: V = 0.257) were observed (Table 3). A binary logistic regression was conducted to examine the effects of sex, age group, residence (rural/urban), hospital stay duration, localization, tumor size, evolution, and treatment on the dependent variable. The full model was not statistically significant, indicating that the predictors did not reliably distinguish between outcome categories overall. Only residence (rural/urban) (*p* = 0.015) and hospitalization length of 8–14 days (*p* = 0.022) were significant, suggesting that these two variables substantially reduced the odds of the outcome.

A series of chi-square tests of independence were conducted to examine the associations between tumor size (2–4 cm) and several clinical variables. There was a significant association between sex and tumor size, *p* = 0.036; the effect size was V = 0.36. Small tumors (<2 cm) were more common in males, whereas medium tumors (2–4 cm) were more frequent in females. A significant relationship was also observed between tumor localization and size, *p* = 0.007; the effect size was V = 0.44. Small tumors were more often found in the right parotid gland, while medium tumors were more commonly located in the left parotid gland. Tumor size was not significantly associated with clinical evolution, *p* = 0.057; the effect size was V = 0.34. The relationship between treatment type and tumor size approached significance but did not reach the conventional threshold, *p* = 0.057; the effect size was V = 0.36. Small- and medium-sized tumors typically underwent total parotidectomy with facial nerve preservation, whereas most of the facial nerve was sacrificed due to the large tumor (Table 4).

### 3.6. Surgical Management

Patients who were cured predominantly underwent partial excision (97.7%). A significant association was observed between treatment type and tumor size, *p* = 0.013; the effect size was V = 0.23. Patients with tumors sized 2–4 cm most frequently underwent partial excision of the parotid gland (56.3%). In contrast, tumors larger than 4 cm were more often managed with total excision of the parotid gland (25.4%). Smaller tumors (<2 cm) were distributed more evenly across treatment modalities, though partial excision remained the most common approach (14.9%).

A chi-square test of independence was conducted to examine the association between sex and treatment type. The results were not statistically significant, *p* = 0.650; the effect size was V = 0.103. Treatment distributions were similar between sexes: both groups most frequently underwent partial excision of the parotid gland. A significant association was also observed between clinical evolution and treatment type, *p* < 0.001; the effect size was V = 0.296 (Table 5).

### 3.7. Postoperative Outcomes

Of the 156 patients in this study, 146 were discharged with the status ‘cured’. One patient with carcinoma ex pleomorphic adenoma was discharged as ‘improved’. Among patients with squamous cell carcinoma metastases, three were discharged as ‘stable’, while one patient, despite disease progression, was discharged at their own request.

### 3.8. Incidental Findings or Unusual Associations

In the examined cohort, four cases of Warthin tumor were identified in association with thyroid colloid goiter. Autoimmune thyroiditis was observed in one individual diagnosed with acinic cell carcinoma and in another with a pleomorphic adenoma. Additionally, two pleomorphic adenomas were found in patients with concurrent malignancies, specifically hepatocellular carcinoma and prostate carcinoma. Two cases of squamous cell carcinoma metastases were linked to underlying viral hepatitis, while chronic hepatitis was associated with one case each of pleomorphic adenoma and basal cell adenoma. Facial nerve paralysis was most frequently observed in conjunction with mucoepidermoid carcinoma, squamous cell carcinoma, and metastatic lesions.

### 3.9. Diagnostic Accuracy (Sensitivity/Specificity)

The sensitivity for malignancy is 70.6%, and specificity of diagnosis is 96.2% (Table 6).

## 4. Discussion

In our study, the incidence of benign tumors was 0.6/100,000 inhabitants, while the incidence of primary malignant tumors of the parotid gland was 0.1/100,000 inhabitants, considering the 25 malignant tumors diagnosed in 5 years in our regional hospital, which serves the population of the northeastern region of Romania (approximately 3.7 million inhabitants, according to the 2021 census). The present study included all benign and malignant cases documented in the pathology department over a five-year period, in contrast with most previous studies that focused on either benign [1,14,26,27] or malignant parotid tumors, such as parotid gland lymphoma [37], parotid acinic carcinoma [31], mucoepidermoid carcinoma [18], squamous cell carcinoma [30], intraparotid metastasis [23], and carcinoma ex pleomorphic adenoma [32]. To date, no incidence of parotid gland tumors has been established in Romania.

Research in the field found that tumors of the parotid gland are infrequent. Despite being thoroughly studied from both morphological and immunohistochemical perspectives, information regarding the incidence of parotid tumors is different in various studies. Some authors included all salivary gland tumors, both benign and malignant neoplasms, estimating a prevalence that varies from 1 per 100,000 individuals in Italy [20] to 5.5 per 100,000 individuals in the United States [38], while other researchers focus solely on tumors of the parotid gland, asserting that parotid gland cancers are rare, representing approximately 0.5% of all cancer cases [8]. Over half a century ago, spanning from 1939 to 1973, a comprehensive analysis of 35 years of clinical experience from the United States encompassed 2807 cases of salivary gland tumors; among these, 1529 cases (54%) were identified as benign tumors, including 1342 cases of benign parotid tumors, while malignant salivary neoplasms were observed in 1278 patients, comprising 354 cases of malignant parotid tumors [38].

The malignancies of major salivary glands are classified as uncommon, as per recent criteria established by the European Society for Medical Oncology (ESMO) and the RARECARE initiative, which stipulates that a rare cancer is characterized by an incidence rate of fewer than 6 cases per 100,000 individuals annually [39]. Malignancies of the salivary glands were considered rare neoplasms constituting 1–5% of total head and neck cancers [40], with the annual incidence of malignant salivary gland tumors approximated at one per 100,000 individuals [41]. A recent study found that the incidence of major salivary gland malignant tumors in Japan is 1.7 per 100,000 inhabitants [39].

Only a few studies include demographic, clinical, and histopathological data on either benign or malignant or both benign and malignant parotid gland tumors [16,42]. A recent study aimed to perform a comprehensive analysis of salivary gland tumors over a five-year period, aiming to delineate their demographic, clinical, and histopathological characteristics and contribute to refining this diagnostic approach in this field. They found that the gender disparities observed in tumor distribution illuminate the complexities of tumor biology and emphasize the need for tailored clinical approaches based on demographic factors [16]. More clinical studies are needed due to the heterogeneous nature of these lesions, which exhibit intricate clinical and pathological characteristics alongside diverse biological behaviors [41], and both clinical and histological diagnostic differentiation between benign and malignant parotid tumors presents significant challenges [17].

The overall benign-to-malignant tumor ratio in our study was 2.6:1, which contrasts with the ratio of 1.4:1 reported by da Silva et al. [3]. We also analyzed in this study whether statistically significant differences are present across different histological subtypes of benign and malignant tumors in terms of ages, tumor size, and surgical methods. A previous study from the literature analyzed the differences between benign and malignant parotid tumors; they found significant differences in terms of ages, tumor size, and surgical methods [43].

Our five-year analysis confirms that pleomorphic adenoma is the most prevalent benign tumor of the parotid gland in our region, followed by Warthin tumor, a finding that aligns with the conclusions of previous research of da Silva [3] and others. In our five-year analysis, pleomorphic adenomas presented at a mean age of 50 years (range: 13–82), consistent with previous studies [1,3,12,44], though at a slightly higher rate than that reported by Abu Ghanem et al. [10]. Warthin tumors occurred at a higher mean age (57 years), also in line with the literature [3,14]. Older patient age has been suggested as a factor contributing to pleomorphic adenoma recurrence [12]. Gontarz et al. [35] compared pleomorphic adenoma, Warthin tumor, and other benign salivary gland tumors, reporting significant age-related differences across groups (0–19, 20–59, ≥60 years) but no significant differences in the parotid gland [35]. In contrast, our study focused exclusively on the parotid gland and included pleomorphic adenoma, Warthin tumor, and basal cell carcinoma. Using narrower age intervals (0–20, 21–30, 31–40, 41–50, 51–60, 61–70, 71–80, 81–90 years), we identified significant differences between groups (*p* = 0.002).

A notable finding in our five-year analysis is that the most frequent primary malignant tumor was acinic cell carcinoma (24% of malignant tumors, 3.8% of all tumors), consistent with the findings of [45]. This contrasts with multiple prior studies that cite mucoepidermoid carcinoma (31% [46] or 50% [18]), primary squamous cell carcinoma (17.6%) [47], or salivary gland carcinoma (23%) [48] as the most common malignancies, suggesting potential regional differences in tumor epidemiology or referral bias in our institution. We found that the incidence of parotid gland metastasis from a neighborhood area was 16.6%, contrary to a recent study performed by Tsang et al. (2024), who found only 6–8% of metastatic neoplastic lesions [23]. As our area is predominantly agricultural, exposure to the sun is greater, and patients develop squamous cell carcinomas and melanomas of the face, which frequently progress to metastases, including at the parotid level, thus explaining the high incidence of parotid metastases in our study.

Interestingly, in our study, benign tumors and acinic cell carcinoma were more common among urban residents, while malignant tumors were more frequent in patients from rural areas, maybe due to different levels of accessibility to medical services. This disparity may reflect differences in healthcare accessibility, screening availability, or socioeconomic factors affecting timely diagnosis and treatment.

This study also evaluated the sensitivity and specificity of preoperative clinical diagnosis in predicting the malignant nature of parotid tumors, which were found to be 56% and 96.93%, respectively.

Notably, the present study is distinctive in that it compares pleomorphic adenoma, Warthin tumor, and basal cell adenoma, revealing significant differences in age distribution (*p* = 0.002), gender (*p* < 0.001), and type of surgical intervention (*p* = 0.016). Park (2012) and Liu (2014) compared recurrent and nonrecurrent pleomorphic adenomas and reported no significant differences between the groups with respect to age (younger or older than 42 years) or tumor size [26], and in terms of gender or mean age [27]. In the present study, only a single case of recurrent pleomorphic adenoma was identified—a 32-year-old female patient with a tumor measuring approximately 3 cm at its greatest dimension, exhibiting a discontinuous capsule and one satellite nodule—thus precluding a meaningful comparison between recurrent and nonrecurrent cases.

The present study showed that age plays a significant role in determining surgical treatment, with younger patients (<50 years) more likely to undergo partial excision, while older patients (≥60 years) demonstrate higher rates of total excision.

Gender distribution of parotid gland tumors is variable, depending on histological subtype. Our data found that pleomorphic adenomas were more frequent in female patients, similar to the findings of other studies [10,12,44]. Warthin tumors showed male predominance, in line with prior studies [14]. In our study, the male-to-female ratio for Warthin tumors was 11.5:1, whereas for pleomorphic adenomas, it was 1:1.5, consistent with the findings of [45]. Gontarz et al. [35] examined gender differences among pleomorphic adenoma, Warthin tumor, and other benign tumors, identifying significant disparities across all salivary glands except the parotid gland [35]. In our cohort, gender differences among the three benign tumor types were statistically significant (*p* < 0.001). Adenocarcinoma NOS appeared more frequently in females in our study, similar with a study conducted by Cunha et al. (2023) on salivary gland tumors [49]. Carcinoma subtypes such as adenoid cystic, acinic cell, and mucoepidermoid carcinoma also showed gender-based variation. Our data support a female predominance, similar to previous reports of female predominance for adenoid cystic carcinoma of salivary glands [31,45,50]. Interestingly, carcinoma ex pleomorphic adenoma was more frequent in females in our studied group, differing from another study in which there was a male predominance [32], likely due to the limited number of cases in our studied group. Gontarz et al. [35] investigated gender-related differences among adenoid cystic carcinoma, mucoepidermoid carcinoma, and other malignant tumors, reporting statistically significant disparities [35].

Benign tumors had a mean size of 3.49 cm in our study, within the commonly reported 2–4 cm range [51]. Warthin tumors had a mean size of 3 cm in our study, consistent with Su et al. (2010) [52]. Grasso et al. [12] showed a high risk of recurrence in the case of pleomorphic adenoma, with a size larger than 2 cm [12]. In our study, the only recurrence of pleomorphic adenoma identified was 3 cm in diameter. Malignant tumor size in our study was generally larger than reported elsewhere [53], with malignant tumors often exceeding 4 cm (mean 4.36 cm), supporting the hypothesis of delayed presentation. Consistent with previous findings [26], the majority of tumors in our study measured between 2 and 4 cm in size. This contrasts with the study conducted by Grasso et al. [12], where the majority of malignant tumors were smaller than 2 cm [12]. This discrepancy may be attributed to differences in the size of the study populations. In their study, Rajbhar et al. (2025) showed significant differences between benign and malignant tumors in terms of tumoral size (*p* = 0.03) [16]. No significant differences were encountered between the benign tumors in our study.

Significant associations were observed between tumor size categories (<2 cm, 2–4 cm, and >4 cm) and gender (*p* = 0.040), laterality (right/left) (*p* = 0.007), type of surgical intervention (*p* = 0.039), and clinical outcome (*p* < 0.001).

The mean age of onset for malignant tumors in the present investigation was determined to be 65 years, which exceeds the mean age associated with benign tumors and aligns closely with the findings of Busch et al., who reported a mean age of 60 years [48], as well as the research conducted by Xiao et al. [46], which indicated a mean age of 61 years, and the study by Guntinas-Lichius et al. [47], where the mean age was recorded at 67 years [46,47]. In the literature, increased age is associated with an increased risk of poor survival [18]. In contrast, acinic cell carcinoma occurred at a mean age of 50 years in our study, which is slightly younger than in some other studies [54], possibly reflecting our smaller sample size. Similarly, carcinoma ex pleomorphic adenoma had a mean age of 61 years in our study, matching previous reports [32]. Adenocarcinoma NOS occurred at a mean age of 66 years in our study, higher than the 57 years reported by da Silva et al. [3]. Squamous cell carcinoma and mucoepidermoid carcinoma were exclusively seen in males at a mean age of 67 years and 80 years, respectively, similar with the male predominance in the case of squamous cell carcinoma reported by Xiao et al. (2021), who found a 72% rate of male distribution [30], and in contrast with the slight female predominance of mucoepidermoid carcinoma found in another study [18]. The low number of malignant tumors in our study makes it almost impossible for an adequate comparison to be made with other studies. Metastatic parotid tumors, particularly from squamous cell carcinoma, predominantly affected older male patients (mean age ~70), in line with the findings of other studies [23,35]. Unlike other studies [37], notably in our study, the mean age of lymphoma onset was 79 years, which is higher than the age reported by [37]. There was a slight female predominance, which contrasts with the male predominance reported in this previous study.

Surgical management in our cohort followed the established protocols. Partial parotidectomy was predominantly used for benign tumors, adenocarcinoma, and lymphoma. In contrast, total parotidectomy—with or without facial nerve preservation—was the preferred strategy for most malignant tumors, depending on the extent of the tumor.

### 4.1. Clinical Implications

The observation that some tumors occur more frequently in patients residing in rural areas, consequently leading to progression differences within this patient group, might be explained by differences in environmental exposures (including smoking rates, levels of pollution, and occupational risks), accessibility to healthcare services, or delayed medical presentation among rural populations.

From a clinical management perspective, these findings underscore the need for targeted strategies to be developed for rural populations, such as improving referral pathways, providing transportation support, and implementing telemedicine or community-based care programs. Such system-level interventions might have the potential to reduce disparities and improve prognosis more effectively than focusing exclusively on tumor-related characteristics.

The length of hospitalization was closely linked with surgical treatment type, with more extensive surgical procedures associated with prolonged durations of hospital stays. These findings reinforce the importance of careful preoperative planning and patient counseling, particularly in older individuals or those undergoing total excision, as they may require longer recovery periods.

### 4.2. Limitations

This study has several limitations. First, the small number of cases in some types of malignant tumors (e.g., one case of basal cell adenocarcinoma and two cases each of myoepithelial carcinoma, mucoepidermoid carcinoma, and squamous cell carcinoma) limits the ability to perform meaningful comparisons between groups and constrains generalizability. Second, this study was conducted over a relatively short period and within a single medical center. Although this institution serves a large proportion of the national population, the findings may not be representative of the overall situation in Romania.

Because this study was retrospective and based primarily on an electronic database without direct patient contact, data on potential risk factors such as smoking or mobile phone use were not available, so we were unable to interpret whether smoking or mobile phone use could have been important risk factors for these tumors. Similarly, information on recurrence, complications, or functional outcomes (e.g., facial nerve function, quality of life) was not collected.

The single-regional hospital design further limits the generalizability of the results to broader populations with different healthcare structures or cultural contexts. In addition, socioeconomic variables, comorbidities, and healthcare access, which may strongly influence diagnostic and treatment distributions, were not included. Finally, moderate interobserver agreement suggested that some patients could receive different clinical judgments depending on the assessor, potentially affecting clinical decision-making.

There is a need to integrate molecular, radiological, and lifestyle risk factors into predictive models to better stratify patients preoperatively. Research should place greater emphasis on modifiable risk factors to guide prevention strategies and enable more targeted screening. Larger, multicenter studies are needed to validate the current findings, detect subtler associations, and facilitate their translation into standardized clinical practice guidelines.

Future research should aim to connect clinical variables and treatment choices with long-term outcomes such as recurrence, functional morbidity, and quality of life following different treatments. Despite advances in tumor characterization, treatment selection is not yet fully individualized. The development of standardized treatment guidelines based on diagnosis, tumor type, and patient comorbidity profiles could improve efficiency and reduce overtreatment. Future research should also assess minimally invasive and nerve-sparing approaches to optimize both oncologic safety and postoperative functional quality of life for patients with parotid gland tumors.

To ensure reliability, studies must include larger cohorts, particularly older patients and those receiving less-common treatments. Prospective or longitudinal designs are essential to clarify causal relationships between demographic characteristics, disease presentation, and treatment approach. By addressing these gaps, clinicians will be better positioned to refine individualized treatment algorithms, ultimately improving outcomes for patients with parotid gland tumors.

## 5. Conclusions

This retrospective study highlights differences in gender, tumor size, and surgical approach between benign and malignant parotid gland tumors. Although it included few malignant cases, it contributes valuable insights into diagnostic accuracy and treatment patterns.

## Figures and Tables

**Table 1:** Demographic characteristics of patients with parotid tumors and types of surgical interventions (n = 221).

		Benign (n = 161) No. (%)	Malignant (n = 60) No. (%)
Onset age (years)	0–20	6 (3.7)	0 (0)
	21–30	11 (6.8)	0 (0)
	31–40	23 (14.3)	1 (1.7)
	41–50	32 (19.9)	4 (6.7)
	51–60	32 (19.9)	16 (26.7)
	61–70	35 (21.7)	15 (25)
	71–80	19 (11.8)	16 (26.7)
	81–90	3 (1.9)	7 (11.7)
	>91	0 (0)	1 (1.7)
Gender	Male	73 (45.3)	44 (73.3)
	Female	88 (54.7)	16 (26.7)
Residence	Rural	71 (44)	34 (56.7)
	Urban	90 (56)	26 (43.3)
Surgery	Biopsy of the salivary gland or duct	8 (5)	6 (10)
	Total parotidectomy with facial nerve resection sacrifice	8 (5)	12 (20)
	Total parotidectomy with facial nerve preservation	27 (16.7)	10 (16.7)
	Partial parotidectomy	96 (59.6)	8 (13.3)
	Lymph node biopsy	2 (1.2)	1 (1.7)
	Radical excision of cervical lymph nodes	1 (0.60)	0 (0)
	Other procedures	19 (11.8)	23 (38.3)

**Table 2:** Clinicopathological characteristics of benign tumors.

Clinicopathological Criteria		Pleomorphic Adenoma (n = 74) No. (%)	Basal Cell Adenoma (n = 8) No. (%)	Warthin Tumor (n = 23) No. (%)	*p* Value *
Age groups (years)	0–20	4 (5.4)	0 (0)	0 (0)	*p* = 0.014
	21–30	8 (10.8)	0 (0)	0 (0)	
	31–40	15 (20.3)	0 (0)	2 (8.7)	
	41–50	13 (17.6)	1 (12.5)	5 (21.7)	
	51–60	19 (25.7)	0 (0)	5 (21.7)	
	61–70	11 (14.9)	5 (62.5)	7 (30.4)	
	71–80	4 (5.4)	1 (12.5)	4 (17.4)	
	81–90	0 (0)	1 (12.5)	0(0)	
Gender	Male	31 (41.9)	4 (50)	21 (91.3)	*p* < 0.001
	Female	43 (58.1)	4 (50)	2 (8.7)	
Surgery	Partial parotidectomy	49 (66.2)	5 (62.5)	16 (69.6)	*p* = 0.054
	Total parotidectomy with facial nerve preservation	18 (24.3)	0 (0)	3 (13)	
	Total parotidectomy with partial facial nerve sacrifice	2 (2.7)	2 (25)	0 (0)	
	Other procedures	5 (6.8)	1 (12.5)	4 (17.4)	

* Chi-square test: *p* < 0.05 is significant.

**Table 3:** Clinicopathological characteristics of malignant tumors.

Clinicopathological Criteria		Primary Tumors (n = 25) No. (%)	MTS (n = 26) No. (%)	*p* Value *
Onset age, years	11–20	1 (4)	0 (0)	*p* = 0.811
	31–40	1 (4)	3 (11.5)	
	41–50	1 (4)	1 (3.8)	
	51–60	5 (20)	4 (15.4)	
	61–70	8 (32)	5 (19.2)	
	71–80	6 (24)	9 (34.6)	
	>80	3 (12)	4 (15.4)	
Gender	Male	12 (48)	15 (57.7)	*p* = 0.488
	Female	13 (52)	11 (42.3)	
Residence	Rural	16 (64)	15 (57.7)	*p* = 0.645
	Urban	9 (36)	11 (42.3)	
Size	<2 cm	2 (8)	7 (26.9)	*p* = 0.170
	2–4 cm	9 (36)	5 (19.2)	
	>4 cm	14 (56)	14 (53.8)	
Surgery	Partial parotidectomy	10 (40)	7 (26.9)	*p* = 0.401
	Total parotidectomy with facial nerve preservation	4 (16)	3 (11.5)	
	Total parotidectomy with facial nerve sacrifice	5 (20)	11 (42.3)	
	Other procedures	6 (24)	5 (19.2)	
Evolution	Aggravated, discharged upon request	0 (0)	1 (3.8)	*p* = 0.289
	Improved	1 (4)	3 (11.5)	
	Stationary	1 (4)	3 (11.5)	
	Healed	23 (92)	19 (73.1)	

* Chi-square test: *p* < 0.05 is significant. MTS: metastasis.

**Table 4:** Analysis of malignant tumor by size.

		Malignant Tumor Size			*p* Value *
		<2 cm No. (%)	2–4 cm No. (%)	>4 cm No. (%)	
Gender	Male	8 (88.9)	5 (35.7)	14 (50)	*p* = 0.036
	Female	1 (11.1)	9 (64.3)	14 (50)	
Location	Right	8 (88.9)	3 (21.4)	13 (46.4)	*p* = 0.007
	Left	1 (11.1)	11 (78.6)	15 (53.6)	
Surgery	Biopsy of the salivary gland or duct	2 (22.2)	0 (0)	1 (3.6)	*p* = 0.057
	Partial parotidectomy	0 (0)	3 (21.4)	4 (14.3)	
	Total parotidectomy with facial nerve preservation	5 (55.6)	7 (50)	5 (17.9)	
	Total parotidectomy with facial nerve resection sacrifice	1 (11.1)	2 (14.3)	13 (46.4)	
	Other procedures	1 (11.1)	2 (14.3)	5 (17.9)	
Evolution	Aggravated, discharged upon request	0 (0)	0 (0)	1 (3.6)	*p* = 0.052
	Improved	1 (11.1)	0 (0)	3 (10.7)	
	Stationary	3 (33.3)	0 (0)	1 (3.6)	
	Healed	5 (55.6)	14 (100)	23 (82.1)	

* Chi-square test: *p* < 0.05 is significant.

**Table 5:** Analysis of treatment procedures (n = 156).

		Partial Parotidectomy No. (%)	Total Parotidectomy with Facial Nerve Preservation No. (%)	Total Parotidectomy with Facial Nerve Sacrifice No. (%)	Other Procedures No. (%)	*p* Value *
Gender	Male	46 (52.9)	13 (46.4)	13 (65)	11 (52.4)	*p* = 0.650
	Female	41 (47.1)	15 (53.6)	7 (35)	10 (47.6)	
	Total	87 (100)	28 (100)	20 (100)	21 (100)	
**Size**	<2 cm	13 (14.9)	5 (17.9)	1 (5)	3 (14.3)	*p* = 0.13
	2–4 cm	49 (56.3)	14 (50)	4 (20)	8 (38.1)	
	>4 cm	25 (28.7)	9 (32.1)	15 (75)	10 (47.6)	
	Total	87 (100)	28(100)	20 (100)	21 (100)	
Evolution	Aggravated, discharged upon request	0 (0)	0 (0)	1 (5)	0 (0)	*p* < 0.001
	Improved	1 (1.1)	0 (0)	3 (15)	0 (0)	
	Stationary	1 (1.1)	0 (0)	0 (3.6)	4 (19)	
	Healed	85 (97.7)	28 (100)	16 (80)	17 (81)	
	Total	87 (100)	28 (100)	20 (100)	21 (100)	

* Chi-square test: *p* < 0.05 is significant.

**Table 6:** Sensitivity and specificity for clinical diagnosis of malignancy.

		Histopathological Type (n)			
		Benign	Malignant	Total	
Clinical diagnosis	Benign	101	15	116	Sensitivity 70.6% Specificity 96.2%
	Malignant	4	36	40	
	Total	105	51	156	

## Data Availability

All the data are presented in the present article.

## Abbreviations

The following abbreviations are used in this manuscript:

ACC	acinic cell carcinoma
ADC	adenocarcinoma
AdCC	adenoid cystic carcinoma
AJCC	American Joint Committee on Cancer
CA ex PA	carcinoma ex pleomorphic adenoma
G	histological grade
Ly	lymphoma
MTS	metastasis
n	number of cases
NOS	not otherwise specified
*p*	probability
pTNM	pathological tumor node metastasis
SCC	squamous cell carcinoma
SMAS	superficial musculoaponeurotic system
WHO	World Health Organization
